# 4D Flow and 2D PC MRI: impact of volumetric coverage and three-directional velocity encoding on quantification of aortic hemodynamics

**DOI:** 10.1186/1532-429X-18-S1-P357

**Published:** 2016-01-27

**Authors:** Emilie Bollache, Pim van Ooij, Alexander L Powell, James C Carr, Michael Markl, Alex J Barker

**Affiliations:** 1 grid.465264.7Department of Radiology, Northwestern University, Chicago, IL USA; 2 grid.465264.7Department of Biomedical Engineering, Northwestern University, Chicago, IL USA

## Background

The accurate assessment of arterial blood flow is crucial for the diagnosis and management of patients with cardiovascular disease. Our aim was to systematically compare three phase-contrast (PC) MRI sequences for the quantification of aortic hemodynamics, including: 1) 2D time-resolved (CINE) PC MRI with one-directional through-plane (2D-1dir) velocity encoding; 2) 2D CINE PC MRI with three-directional (2D-3dir) velocity encoding; 3) 4D flow MRI with full volumetric coverage of the aorta and three-directional velocity encoding.

## Methods

**Figure 1:**
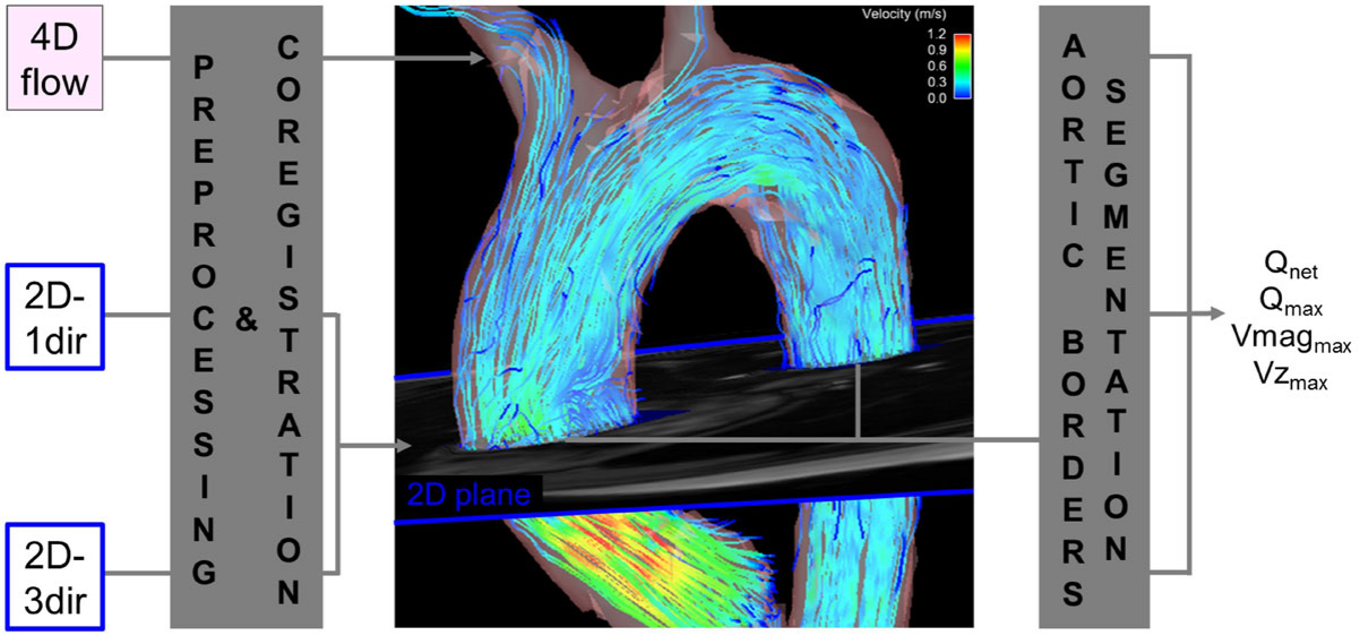
**Workflow for the analysis of PC MRI data, including 1) preprocessing of all 4D flow, 2D-1dir and 2D-3dir images, 2) aortic 3D segmentation from 4D flow data along with positioning of a plane co-registered with the 2D slice coordinates, and 3) 2D segmentation of ascending and descending aortic borders used for the estimation of new volume (Q**_**net**_**), peak flow (Q**_**max**_**) as well as peak velocity magnitude (Vmag**_**max**_**) and through-plane velocity (Vz**_**max**_**)**.

We studied 15 healthy volunteers (51 ± 19 yrs; 11 men) with MRI (1.5T MAGNETOM Aera, Siemens). 4D flow MRI was acquired in a sagittal oblique 3D volume covering the thoracic aorta (free breathing; spatial resolution (sr) = 3.1 × 2.3 × 2.5 mm^3^; temporal resolution (tr) = 39.2 ms). In addition, 2D-1dir (breath-hold; sr = 1.8 × 2.5 × 6 mm^3^; tr = 38.4 ms) and 2D-3dir (free breathing; sr = 1.8 × 2.5 × 6 mm^3^; tr = 38.4 ms) CINE PC MRI were acquired in a 2D plane perpendicular to both the ascending (AA) and descending (DA) aorta. For all acquisitions, the velocity sensitivity (venc) was 150cm/s and prospective ECG gating was used. All 2D CINE PC and 4D flow MRI data were preprocessed to correct for Maxwell terms, velocity aliasing and eddy currents (Figure 1). 4D flow MRI data analysis included the 3D segmentation of the aorta (Mimics, Materialise) with a plane placed orthogonal to the aortic centerline (Ensight, CEI) and coregistered to the scanner coordinates of the 2D slices used for the 2D CINE PC MRI acquisitions. Delineation of the AA and DA vessel lumen contours enabled the measurement of net flow volume (Q_net_), peak flow (Q_max_) and peak velocity at both locations. Peak velocity was calculated using the through-plane component only (Vz_max_) for the 3 sequences, or the Euclidian norm of the three principal directions (Vmag_max_) for 4D flow and 2D-3dir CINE PC MRI.

**Figure:**
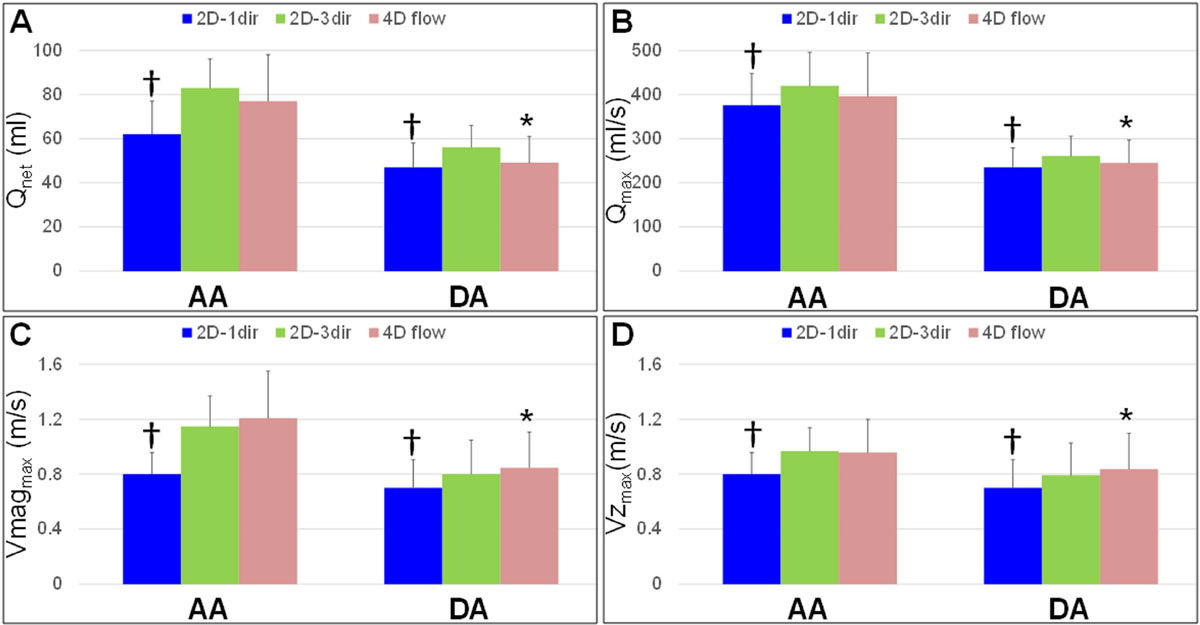
Figure 2

## Results

All 2D-1dir CINE PC MRI indices in both the AA and DA were significantly lower than 2D-3dir indices, while a better agreement was found between 4D flow and 2D-3dir measurements (Figure 2). Differences between 2D-1dir and 2D-3dir indices, in percentage of the mean value, were lower in the DA than in the AA (Table), suggesting that flow is more complex in the AA and three-directional encoding is required to fully capture flow dynamics.

## Conclusions

Our results suggest the importance of three-directional encoding of velocity for estimation of hemodynamic indices, especially when considering volume and peak velocity measured in the AA. Differences between the clinically used 2D-1dir and 2D-3dir CINE PC MRI sequences could also be attributed to changes in aortic hemodynamics between breath-hold and free-breathing acquisitions, highlighting the importance of technique-specific reference values for aortic flow and velocity indices.

